# Numerical Evaluation of Diffraction Integrals

**DOI:** 10.6028/jres.105.048

**Published:** 2000-08-01

**Authors:** Klaus D. Mielenz

**Affiliations:** Oakland, MD 21550

**Keywords:** circular aperture, diffraction, half plane, numerical integration, recursion formula, slit

## Abstract

This paper describes a simple numerical integration method for diffraction integrals which is based on elementary geometrical considerations of the manner in which different portions of the incident wavefront contribute to the diffracted field. The method is applicable in a wide range of cases as the assumptions regarding the type of integral are minimal, and the results are accurate even when the wavefront is divided into only a relatively small number of summation elements. Higher accuracies can be achieved by increasing the number of summation elements and/or incorporating Simpson’s rule into the basic integration formula. The use of the method is illustrated by numerical examples based on Fresnel’s diffraction integrals for circular apertures and apertures bounded by infinite straight lines (slits, half planes). In the latter cases, the numerical integration formula is reduced to a simple recursion formula, so that there is no need to perform repetitive summations for every point of the diffraction profile.

## 1. Basic Equations

**Fig. 1:**
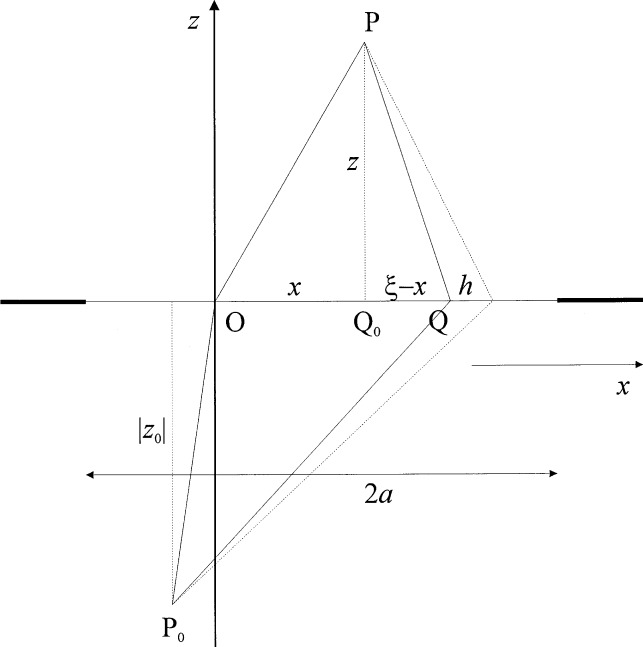
Cross section of a plane diffracting aperture (*xz*-plane).

Diffraction problems that can be described in two dimensions as indicated in Fig. 1 usually lead to complex integrals of the type

U(x,z)=∫dξA(ξ,x,z)B(ξ,x,z), (1)

where *A* and *B* account for the amplitude and phase of the optical field at a point of observation P = (*x*,*z*) due to a point *Q* = (*ξ*,0) located inside a diffracting aperture. In practice, the phase term *B* oscillates rapidly inside the range of integration while the amplitude term *A* varies slowly. The phase term *B* is often, but not always, a sinusoidal function of the form e^i^*^kg^*^(^*^ξ^*^)^, where *k* = 2π/λ is the circular wavenumber of monochromatic light with wavelength λ.

When closed analytical expressions for *U* are not available it is sometimes possible to find approximate solutions by the method of stationary phase [1,2]. However, in this author’s experience, the results obtained can be unreliable. A potential source of error is the basic premise of the method itself; namely, that the rapid oscillations of the phase term *B* nullify each other except in the vicinity of stationary points. This is presumed true on account of the slow variation of the amplitude term *A*, but inconsistent with the well-known fact that the corresponding series encountered in connection with Fresnel’s zone construction, *A*_1_−*A*_2_ + *A*_3_ −…− *A_n_*, has a finite value even when the terms alter their absolute values very gradually [3,4]. Accordingly, substantial errors can occur if the contribution of the stationary points is weak. As shown in Appendix A, the stationary-phase method fails completely when applied to Fresnel diffraction at a slit or half plane.

For these reasons, it may be preferable to use numerical integration. In doing so, the width of the summation elements, ∆*ξ* = *h*, must be sufficiently small to ensure that all oscillations of the phase term *B* are accurately sampled. On the other hand, the computations would be unnecessarily complicated if *h* is too small. A general rule for choosing *h* can be established as follows. As we are dealing with diffraction, the period of the oscillations does not depend on the specific functional form of *B* but only on the path length *P*_0_*Q* + *QP*, where *P*_0_ = (*x*_0_,*z*_0_) is the source point shown in Fig. 1. When the point *Q* is moved along the *x*-axis by an increment *h*, this path length changes by

$$\begin{array}{c} \delta =\sqrt{z_{0}^{2}+{\left(\xi +h-x_{0}\right)}^{2}}-\sqrt{z_{0}^{2}+{\left(\xi -x_{0}\right)}^{2}}\\ \;-\sqrt{z^{2}+{\left(\xi +h-x\right)}^{2}}+\sqrt{z^{2}+{\left(\xi -x\right)}^{2}}\\ \;\approx h\left(\frac{\xi -x_{0}}{\left|z_{0}\right|}+\frac{\xi -x}{z}\right), \end{array}$$ (2)

where it is assumed that (*ξ*−*x*_0_)^2^ ≪ *z*_0_^2^, (*ξ*−*x*)^2^≪*z*^2^, and terms in *h*^2^ are ignored. Hence it follows from the quarter-wave criterion that reliable results can be expected when *h* is chosen so that *δ*<*λ*/4.

**Fig. 2:**
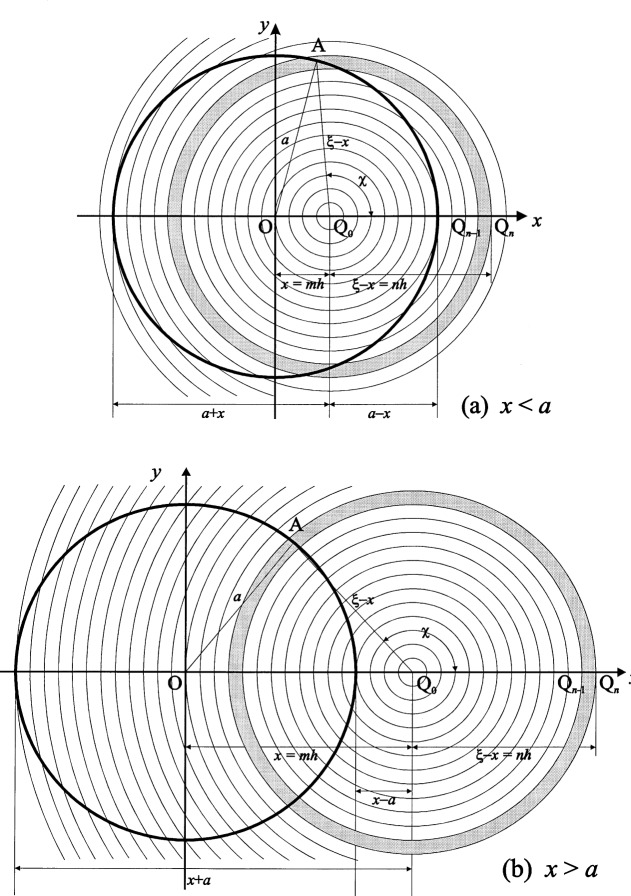
Annular summation elements for a circular aperture (*xy*-plane). (a) Lit region. (b) Shadow region.

We now divide the aperture halfwidth *a* into *N* summation elements bounded by equidistant points Q*_n_*, as illustrated in Figs. 2a, b, and 4, and define

$$OQ_{0}=x=mh,\;Q_{0}Q_{n}=\xi -x,\;Q_{n-1}Q_{n}=h=\frac{a}{N},$$ (3a)

where O is the coordinate origin, Q_0_ is the projection of the point of observation P onto the *x*-axis, and *m*, *n*, and *N* are integers. Accordingly, Eq. (1) can be replaced by the quadrature formula

$$U(x,z)\approx U_{m}=\frac{a}{N}\sum_{n\neq 0}A_{mn}B_{mn},$$ (3b)

where

$$A_{mn}=A\left[\left(n-\frac{1}{2}\right)h,mh,z\right],\;B_{mn}=B\left[\left(n-\frac{1}{2}\right)h,mh,z\right]$$ (3c)

are the values of *A* and *B* at the midpoints of the summation elements and the limits of summation depend on the diffraction problem being considered. The value of *N* to be used in these formulae can be estimated from Eq. (2) by assuming a distant source (|*z*_0_|≫*z*) so that δ ≈ *h*(*ξ*−*x*)/*z* = *a*^2^*n*/(*N*^2^*z*). If this is to be less than *λ*/4 for every summation element used for the computations, a good upper limit for *n* is 3*N*,^1^ and then one finds

$$N\geq \frac{12a^{2}}{\lambda z}=\frac{6u}{\pi }\approx 2u,$$ (3d)

where *u* = *ka*^2^/*z* is the familiar configuration parameter of Fresnel’s diffraction theory for |*z*_0_|≫*z*. As it is well known that diffraction patterns pertaining to large values of *u* are highly structured [4], this result makes good sense in that it stipulates narrower summation elements when *u* increases. The accuracy of the numerical computations can also be improved by replacing the value of *B_mn_* in Eq. (3c) with the value corresponding to Simpson’s rule,

$$B_{mn}=\frac{1}{6}\{B\left[\left(n-1\right)h,mh,z\right]+4B\left[\left(n-\frac{1}{2}\right)h,mh,z\right]+B\left[nh,mh,z\right]\}.$$ (3e)

By means of trial computations, it was found that this substitution can result in a tenfold improvement of accuracy.

The above equations are intended for applications where closed solutions of Eq. (1) cannot be found and will be used in future research. In the remainder of the present paper, their validity will be demonstrated by numerical examples involving the Fresnel diffraction integral

$$U_{F}\left(P\right)=U_{\text{geom}}\left(P\right)\alpha_{F}\left(P\right),\;\alpha_{F}\left(P\right)=\frac{-i}{\lambda z}\int dQe^{ik\left(QP-z\right)},$$ (4a)

where *U*_geom_ is the geometrical field in the absence of diffraction, *α*_F_ is the modification of the field by diffraction, and where

$$QP=\sqrt{{\left(\xi -x\right)}^{2}+\eta^{2}+z^{2}}\approx z+\frac{{\left(\xi -x\right)}^{2}+\eta^{2}}{2z}.$$ (4b)

These expressions are valid in the paraxial Fresnel approximation for a distant source point P_0_ and pertain to a point of observation P = (*x*,0,*z*) as in Fig. 1 whereas the point *Q* = (*ξ*,*η*,0) is assumed to lie anywhere in the aperture plane *z* = 0. In Secs. 2 and 3 of the paper, *α*_F_ will be reduced to a two-dimensional integral for the respective cases of circular apertures and apertures bounded by infinite straight lines (slit and half plane). The results of the numerical integration will be shown and compared to the corresponding exact solutions, which may be found in Ref. [5].

## 2. Circular Aperture

For a circular aperture of radius *a*, Fresnel’s integral in Eq. (4a) can be reduced to a single integral by assuming annular area elements d*Q* which are centered on the projection Q_0_ of the point of observation onto the aperture plane, as indicated in Figs. 2a and 2b. With O as the aperture center, *x* = *OQ*_0_ > 0 and *ξ* = *OQ* > *x* for points to the right of Q_0_, the distance *QP* defined by Eq. (4b) will then be constant and equal to

$$QP=z+{\left(\xi -x\right)}^{2}/2z$$ (5a)

everywhere on d*Q* and the integration can be carried out over *ξ* alone. As the annular area elements are eccentric to the aperture they are, in general, partially obstructed by the aperture rim so that their effective areas will be given by

dQ=2π(1−χ/π)d(ξ−x)(ξ−x), (5b)

where *χ* = ∠AQ_0_Q is the semi-angle subtended at Q_0_ by the intersection of the area elements with the aperture rim, as indicated in the Figs. 2a and 2b.

When Q_0_ lies inside the aperture (*x* ≤ *a*, as in Fig. 2a), the innermost area elements with radii *ξ*−*x*≤*a*−*x* are unobstructed and fully contained in the aperture (*χ* = 0), and the outermost elements with radii *ξ*−*x* > *a* + *x* are fully obstructed (*χ* = π). For the intermediate elements the angle *χ* is found by applying the cosine theorem to the triangle OAQ_0_ shown in the figure, so that

$$\cos\;\chi =\frac{a^{2}-x^{2}-{\left(\xi -x\right)}^{2}}{2x\left(\xi -x\right)}.$$ (5c)

Thus, upon substitution of Eqs. (5a) and (5b) into Eq. (4a) and noting that 2π/(*λz*) = *k*/*z* = *u*/*a*^2^,

$$\begin{array}{c} \alpha_{F}\left(x\right)=\frac{-iu}{a^{2}}[\int_{0}^{a-x}d\left(\xi -x\right)\left(\xi -x\right)e^{ik{\left(\xi -x\right)}^{2}/\left(2z\right)}\\ +\int_{a-x}^{a+x}d\left(\xi -x\right)\left(\xi -x\right)\left(1-\chi /\pi \right)e^{ik{\left(\xi -x\right)}^{2}/\left(2z\right)}]\\ =\left[1-e^{iu{\left(a-x\right)}^{2}/(2a^{2})}\right]\\ -\frac{iu}{a^{2}}\int_{a-x}^{a+x}d\left(\xi -x\right)\left(\xi -x\right)\left(1-\chi /\pi \right)e^{ik{\left(\xi -x\right)}^{2}/\left(2z\right)},\;x\leq a, \end{array}$$ (5d)

where the first integral was reduced to an elementary expression by substituting i*k*(*ξ*−*x*)^2^/2*z* as the integration variable. When Q_0_ lies outside the aperture (*x*≥*a*, as in Fig. 2b), the inner elements with radii *ξ*−*x*≤*x*−*a* and the outer elements with radii *ξ*−*x*≤*x*+*a* are all fully obstructed (*χ* = π). In the intermediate region, Eq. (5c) applies once again^2^ and we have

$$\alpha_{F}\left(x\right)=\frac{-iu}{a^{2}}\int_{x-a}^{x+a}d\left(\xi -x\right)\left(\xi -x\right)\left(1-\chi /\pi \right)e^{ik{\left(\xi -x\right)}^{2}/\left(2z\right)},x\geq a.$$ (5e)

The integrals in Eqs. (5d) and (5e) can now be identified with Eq. (1), with

$$A\left(\xi ,x,z\right)=\frac{-iu\left(\xi -x\right)\left(1-\chi /\pi \right)}{a^{2}},B\left(\xi ,x,z\right)=e^{ik{\left(\xi -x\right)}^{2}/\left(2z\right)},$$ (6a)

so that, according to Eqs. (3a–c),

$$\alpha_{F}\left(P\right)\approx a_{m}=\left[1-e^{iu{\left(N-m\right)}^{2}/\left(2N^{2}\right)}\right]-\frac{iu}{N^{2}}\sum_{n=N-m+1}^{N+m}n\left(1-\chi_{mn}/\pi \right)e^{iu{\left(n-\frac{1}{2}\right)}^{2}/(2N^{2})},\;m\leq N$$ (6b)

$$\alpha_{F}\left(P\right)\approx \alpha_{m}=-\frac{iu}{N^{2}}\sum_{n=m-N}^{m+N}n(1-\chi_{mn}/{\pi )e}^{iu{\left(n-\frac{1}{2}\right)}^{2}/(2N^{2})},$$ (6c)

where

$$\chi_{mn}=\cos^{-1}\left[\frac{N^{2}-m^{2}-{\left(n-\frac{1}{2}\right)}^{2}}{2m\left(n-\frac{1}{2}\right)}\right].$$ (6d)

**Fig. 3:**
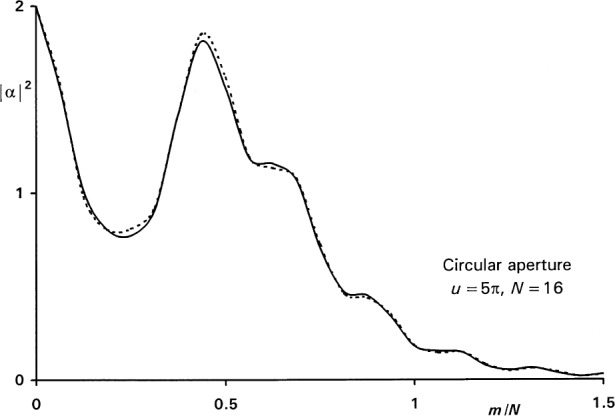
Approximate (—) and exact (----) diffraction profiles of a circular aperture.

The use of these equations on a personal computer is simple. As an example, Fig. 3 compares the numerical values of |*α_m_*|^2^ obtained for *u* = 5π and *N* = 16 to the exact results given by the Fresnel-Lommel theory. The agreement is good and improves when larger values of *N* are used, as indicated in the left-hand column of Table 1. The values listed in the table are the maximum errors encountered in the range *m*≤3*N*. In this particular case they occurred near the center of the profile (*m*≤0.5*N*).

## 3. Apertures Bounded by Infinite Straight Lines

**Fig. 4:**
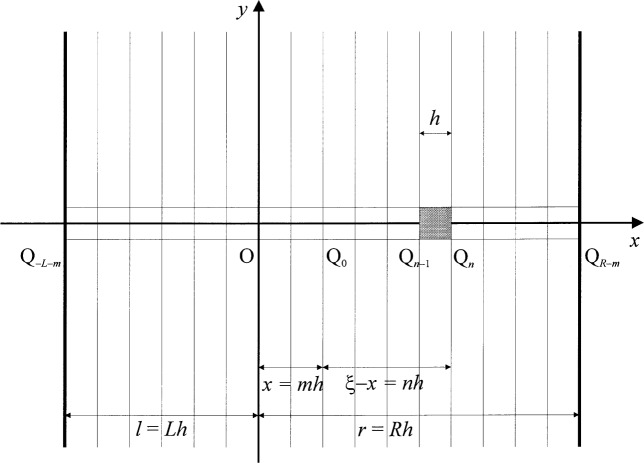
Rectangular summation elements for apertures bounded by straight lines (*xy*-plane).

For a plane aperture of width (*l* + *r*) which is bounded by infinite straight lines as in Fig. 4, the reduction of the integral of Eq. (4a) to two dimensions is readily achieved by choosing cartesian coordinates so that the *y*-axis is parallel to the aperture edges. Letting P = (*x*,0,*z*) as before, this leads to

$$\alpha_{F}\left(P\right)=\frac{-i}{\lambda z}\int_{-\infty }^{\infty }d\eta e^{ik\eta^{2}/2z}\int_{-l}^{r}d\xi e^{ik{\left(\xi -x\right)}^{2}/(2z)}=\frac{-{\mathrm{ie}}^{i\pi /4}}{\sqrt{\lambda z}}\int_{-(l+x)}^{\left(r-x\right)}d(\xi -x)e^{ik{\left(\xi -x\right)}^{2}/(2z)},$$ (7a)

where

$$\int_{-\infty }^{\infty }d\eta e^{ik\eta^{2}/(2z)}=\sqrt{2\lambda z}F\left(\infty \right)=\sqrt{\lambda z}e^{i\pi /4}$$ (7b)

and *F*(∞) = e^iπ/4^ is the complex Fresnel integral at infinity.

The amplitude term *A*(*ξ*,*x*,*z*) in Eq. (1) is now given by the factor $-{\mathrm{ie}}^{i\pi /4}/\sqrt{\lambda z}$ that appears outside the integral of Eq. (7b) so that, on letting *l* = *Lh* and *r* = *Rh* as indicated in Fig. 4 and using Eqs. (3a) to (3d), we find

$$\alpha_{m}=\frac{-ihe^{\mathrm{i\pi}/4}}{\sqrt{\lambda z}}\sum_{n=-(L+m)}^{R-m}e^{ik{\left(n-\frac{1}{2}\right)}^{2}h^{2}/(2z)},\;n\neq 0.$$ (8a)

It follows immediately that, if *α_m_* is known and P is moved to the right or left by ±*h*, the new value of *α_m_* is given by the recursion formula

$$\alpha_{m\pm 1}=\alpha_{m}\mp \frac{ihe^{i\pi /4}}{\sqrt{\lambda z}}[e^{ik{\left(L+m+\frac{1}{2}\right)}^{2}h^{2}/(2z)}-e^{ik{\left(R-m-\frac{1}{2}\right)}^{2}2h^{2}/(2z)}],$$ (8b)

which illustrates in a very instructive manner how the diffraction pattern changes when the point of observation is moved, so that one portion of the wavefront is uncovered by the aperture and another portion is covered. The recursion formula Eq. (8b) is convenient for practical computations as it allows the computation of successive values of *α_m_* without performing the summation of Eq. (8a) for every point. In the examples given below, a starting value for *α_m_* is obtained from known closed solutions, so that there is no need at all to perform a summation. This use of an exact starting value also improves the accuracy of the computations because it forces the initial error to be zero.

### 3.1 Slit

For a slit of width 2*a*, we define *l* = *r* = *a*, *L* = *R* = *N* and *h* = *a*/*N*, so that

$$\frac{h}{\sqrt{\lambda z}}=\frac{1}{N}\sqrt{\frac{u}{2\pi }},\;\frac{kh^{2}}{z}=\frac{u}{N^{2}},$$ (9a)

$$\alpha_{m\pm 1}=\alpha_{m}-\frac{i}{N}\sqrt{\frac{u}{2\pi }}e^{i\pi /4}[e^{iu{\left(N+m+\frac{1}{2}\right)}^{2}/(2N^{2})}-e^{iu{\left(N-m-\frac{1}{2}\right)}^{2}/(2N^{2})}],$$ (9b)

where it is noted that it is sufficient to perform the computations for *m* > 0 as the diffraction pattern is symmetrical.

On account of the trigonometric identity

$$e^{i\alpha }-e^{i\beta }=e^{i(\alpha +\beta )/2}[e^{i(\alpha -\beta )/2}-e^{-i(\alpha -\beta )/2}]=2{\mathrm{ie}}^{i(\alpha +\beta )/2}\;\sin[(\alpha -\beta )/2],$$ (10a)

this can be transformed into the following expressions, which are convenient for practical computations:

$$\alpha_{m+1}=\alpha_{m}+\frac{1}{N}\sqrt{\frac{2u}{\pi }}e^{iX_{m}}\sin Y_{m},$$ (10b)

$$X_{m}=\frac{\pi }{4}+\frac{u}{2}\left[1+\frac{{\left(m+\frac{1}{2}\right)}^{2}}{N^{2}}\right],\;Y_{m}=\frac{u\left(m+\frac{1}{2}\right)}{N}.$$ (10c)

**Fig. 5:**
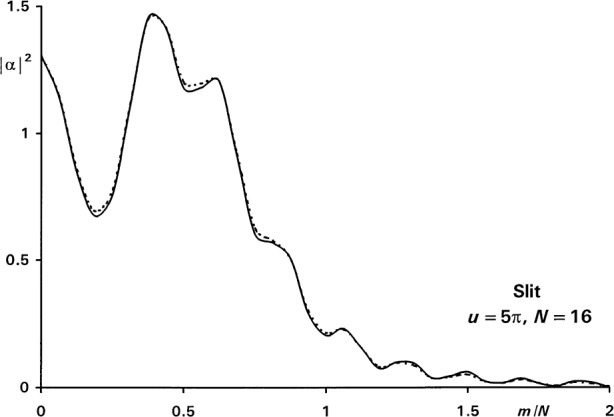
Approximate (—) and exact (----) diffraction profiles of a slit.

As an example, Fig. 5 shows the approximate and exact diffraction profiles of a slit for *u* = 5π, *N* = 16, and using the well-known Fresnel solution, $\alpha_{F}\left(0\right)=(1-i)F(\sqrt{u/\pi })$, as the starting value. The two curves resemble each other closely, the largest errors being on the order of 0.023 and occurring near *m*/*N* = 0.5. The improvement of accuracy achieved by using larger values of *N* is shown in the center portion of Table 1.

### 3.2 Half Plane

On letting *L* = 0 and *R* = ∞, Eqs. (8a) and (8b) apply to a diffracting straight edge which coincides with the *y*-axis of Fig. 4. As there is no aperture edge on the right, the last term of Eq. (8b) is now absent and one obtains

$$\alpha_{m\pm 1}=\alpha_{m}\mp \frac{ihe^{i\pi /4}}{\sqrt{\lambda z}}e^{ik{\left(m\pm 1-\frac{1}{2}\right)}^{2}h^{2}/(2z)}.$$ (11a)

The previous definition of *h* as a given fraction of aperture width is no longer applicable but can be replaced by taking *h* as a certain fraction, say $h=\sqrt{\lambda z}/M$, of the width of the first Fresnel zone. Hence it follows easily that

$$\alpha_{m\pm 1}=\alpha_{m}\mp \frac{i}{M}e^{iX_{m\pm 1}},\;X_{m\pm 1}=\pi \left[\frac{1}{4}+\frac{{\left(m\pm 1-\frac{1}{2}\right)}^{2}}{M^{2}}\right].$$ (11b)

**Fig. 6:**
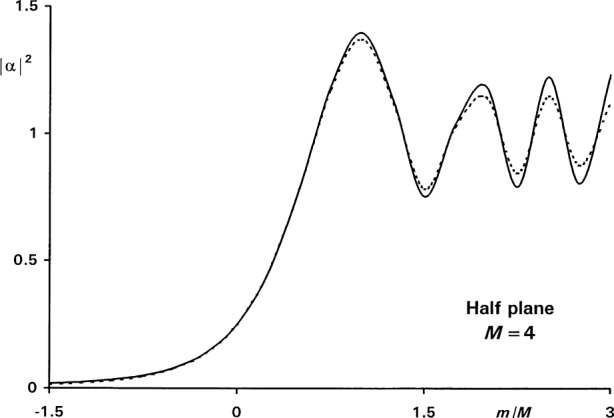
Approximate (—) and exact (----) diffraction profiles of a half plane.

Figure 6 shows the diffraction pattern computed in this manner for *M* = 4, using the well-known Fresnel solution, *α*_F_(0) = ½, as the starting value. The agreement with the exact result is within ±0.025 for − 4 ≤ *m*/M < 1, but considerably worse beyond these limits. These discrepancies are reduced when larger values of *M* are used, as may be seen from the right-hand side of Table 1.

## Figures and Tables

**Table 1:** The largest errors in diffraction profiles computed from Eqs. (6a), (6b), (6c), (10a), (10b), and (11b).

*N*	Circular aperture (*u*=5π)	Slit (*u*=5π)	*M*	Half plane
16	6.0×10^−2^	2.3×10^−2^	8	1.9×10^−2^
32	2.3×10^−2^	5.6×10^−3^	16	4.3×10^−3^
64	6.4×10^−3^	1.4×10^−3^	32	1.1×10^−3^
128	2.5×10^−3^	3.6×10^−4^	64	2.7×10^−4^
256	9.6×10^−4^	9.0×10^−5^	128	6.6×10^−5^
512	3.5×10^−4^	2.3×10^−5^	256	1.7×10^−5^

## Footnotes

1 The value pertains to a point of observation located one aperture halfwidth beyond the shadow boundary. A larger value should be used if necessary.

2 Although *χ* is discontinuous on crossing the shadow boundary, Eqs. (5d) and (5e) are equivalent and give identical results for *x* = *a*.

## 4. Appendix A. Evaluation of Eq. (7a) by the Method of Stationary Phase

Equation (7a) is of the form

$$\alpha_{F}\left(x\right)=\int_{-(l-x)}^{\left(r-x\right)}d\phi Ae^{ikg(\phi )},$$

where *π* = *ξ*−*x*, $A=-ie^{i\pi /4}/\sqrt{\lambda z}$, *g*(*ϕ*) = *ϕ*^2^/*z*. The only stationary point, defined by *g*′(*ϕ*) = *ϕ*/*z* = 0, occurs at *π*_0_ = 0 so that, according to the Appendix of Ref. [2], the stationary-phase value of the integral is

$$\alpha_{F}\left(x\right)\approx \frac{\sqrt{\lambda }\varepsilon }{\sqrt{\left|g^{\prime \prime }\left(\phi_{0}\right)\right|}}Ae^{ikg(\phi_{0})},$$

where *g*″ (*ϕ*_0_) = 1*/z* and *ε* = e^+iπ/4^ according as *g″* (*ϕ*_0_) is positive or negative. Since 1/*z* is positive, this gives *α*_F_ ≈ 1 so that the “diffraction pattern” of a slit or half plane would be identically equal to one everywhere in the plane of observation.
